# Cone-Beam Computed Tomography (CBCT)-Based Diagnosis of Dental Bone Defects

**DOI:** 10.3390/diagnostics14131404

**Published:** 2024-07-01

**Authors:** Faisal Alshomrani

**Affiliations:** Department of Diagnostic Radiology Technology, College of Applied Medical Science, Taibah University, Medinah 41477, Saudi Arabia; fshomrani@taibahu.edu.sa

**Keywords:** computed tomography, dental imaging

## Abstract

Cone Beam Computed Tomography (CBCT) has completely changed the way that bone disorders are diagnosed and treated, especially in the dental and maxillofacial domains. This article examines the diverse applications of computed tomography (CBCT) in the diagnosis and treatment of facial trauma, including mandibular, dentoalveolar, and other facial fractures, as well as bone abnormalities like dislocations and fractures. CBCT is useful for a wide range of dental conditions and greatly improves diagnostic accuracy in periodontics, orthodontics, endodontics, and dental implantology. Additionally, a comparison between CBCT and conventional imaging methods was conducted, emphasizing the latter’s inferior 3D imaging capabilities, allowing for more precise treatment planning and better patient outcomes with CBCT. Although CBCT has many benefits, it also has some drawbacks, such as requiring specific training for accurate interpretation, cost considerations, and a higher radiation exposure than with traditional dental X-rays. In order to optimize benefits and reduce risks, the conclusion highlights CBCT’s revolutionary influence on clinical practice while arguing for its prudent and responsible application.

## 1. Introduction

**Figure 1:**
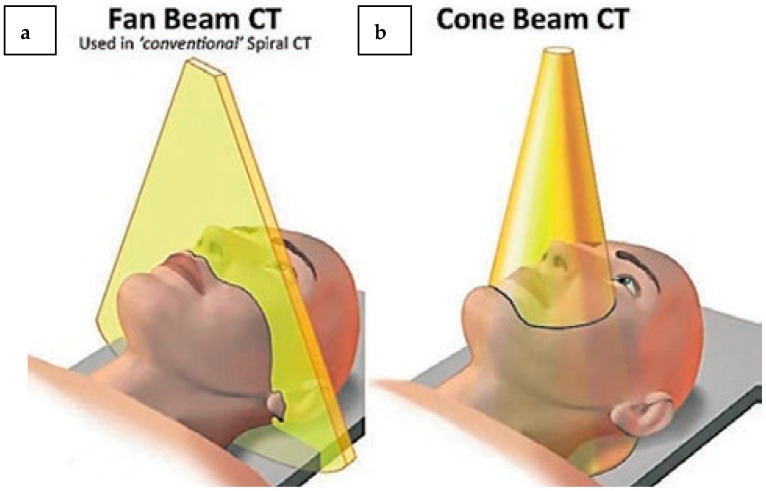
Description of principle of CBCT in comparison to conventional CT. (**a**) In CBCT, the gantry rotates around the patient just once before the cone-shaped X-ray beam arrives at a flat detector. (**b**) Multiple linear detectors and a narrowly collimated, fan-shaped beam revolve around the patient in a conventional CT scan to collect multiple image portions per rotation. Volumetric images are converted into a three-dimensional volume dataset of photos using both methods. Published with permission from [5]. Copyright 2006, Elsevier.

Advancements in technology have propelled the medical field to unprecedented heights and allowed for numerous achievements across multiple specialties, chief among them, radiology. In the field, computed tomography (CT), radiography, ultrasound, MDCT (Multidetector Computed Tomography), and magnetic resonance imaging (MRI) are the most often-used modalities [1,2]. CT imaging is the best technique for imaging changes in bone, even though radiography is widely available and MRI offers excellent soft tissue contrast [3]. The primary drawback of the CT scan is the high radiation dosage it exposes the patient to; as a result, its application in the orofacial region is restricted. Due to the CT scan’s limitations, a device that offers all of the benefits of the CT scan without any of the drawbacks has been developed. For the patient, this essentially means a lessened level of radiation. The orofacial region can be seen in three dimensions using Cone-Beam Computed Tomography (CBCT), in accordance with the ALARA principle (As Low as Reasonably Achievable). With just one spin of the X-ray beam and detector, CBCT can cover the scanned area thanks to a cone-shaped X-ray radiation pattern (Figure 1) [4].

Clinical CT imaging devices are useful for joint trauma imaging, radiotherapy planning, orthopaedic, and dental pathologies with voxel sizes up to 100–200 µm [6,7]. For instance, it has been established that CBCT is the best modality to use when evaluating wrist fractures [8]. The apparent clinical characteristics in CBCT can only be 500 µm in size because, despite the resolution mentioned, the actual spatial resolution is roughly twice lower according to Nyquist’s theorem [9]. Nevertheless, this is insufficient to detect microstructural changes in the bone. The focal spot size of the beam, motion, receptor size, acquisition geometry, and radiation dose all have an impact on the quality of the CBCT images. Test objects that simulate tissue, or quality assurance phantoms, make it possible to evaluate the operational image resolution of a CT machine. The spatial resolution of clinical CT can be measured using the modulation-transfer function (MTF) or task-transfer function [10]. The resolution limit is roughly seven line pairs per centimeter [11]. In actuality, the MTF can be estimated using a high-contrast edge or a sequence of line pair patterns. Detectability of low contrast, consistency, noise power spectrum, and precision of CT numbers are additional parameters related to CT image quality [12]. This review highlights the significance and current research status of the CBCT technique in diagnosis of dental bone defects, its advantages, and comparison to the traditional techniques for bone defect diagnosis, arguing for its diagnostic efficiency and accuracy.

The formerly published reviews have not highlighted the new findings, or they have discussed the role of the CBCT technique in diagnosis of specific bone abnormalities like mandibular, dental, and alveolar bone defects. This review was compiled by reviewing past literature on diagnosis of dental bone defects by CBCT from ScienceDirect, PubMed, Scopus, Web of Science, and others. Our study focuses on the research on CBCT for diagnosis of dental bone defects for approximately the last decade.

## 2. Role of CBCT in Bone Anomalies

### Bone Dislocations and Fractures

**Figure 2:**
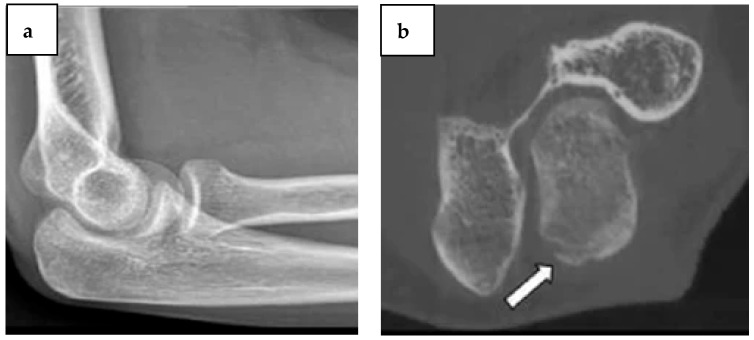
Ulna olecranon occult. (**a**) The elbow’s lateral view CR reveals no signs of a fracture. (**b**) An axial CBCT reorganized image at the posterior part of the olecranon process shows an obscure adjacent bone fragment and a subtle disruption of the cortical structure pointed with the white arrow. Published with permission from [16]. Copyright 2015, Springer Nature.

Computed Radiography (CR) is the primary imaging method when there is clinical concern of fractures, despite its lower sensitivity compared to cross-sectional imaging. Compared to CR, CBCT imaging is more sensitive in identifying minor bone and joint trauma. It can also confirm suspicious fractures or show fractures that are invisible on CR (Figure 2). This affects the treatment plan in the majority of cases [13]. When CBCT and MDCT, the gold standards for diagnosing finger fractures, are compared, similar findings are obtained in terms of articular involvement assessment and fracture visualization [14]. When there is a substantial likelihood of carpal fractures, particularly involving the scaphoid bone, but there is no confirmed fracture, a follow-up MRI is frequently advised to rule out occult nondisplaced ruptures and bone marrow concussion. MRI is still more responsive than CBCT in this situation [15].

**Figure 3:**
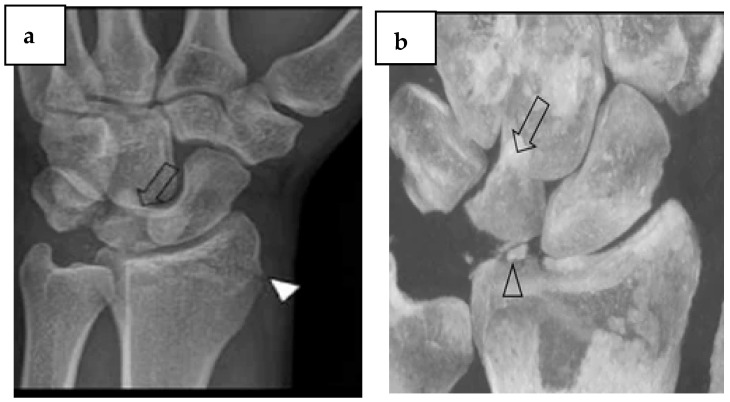
Complex fracture involving multiple intra-articular fracture fragments, perilunate dislocation, and involvement of the dorsal side of the radius’s styloid process. (**a**) The distal radius fracture (arrowhead) and perilunate dislocation (arrow) are visible in the CR (oblique view). (**b**) 3D reconstruction of CBCT images helpful in assessing the carpal bone displacement (open arrow) and the presence of further fracture fragments (open arrowhead). Published with permission from [18]. Copyright 2018, Springer Nature.

However, due to possible contraindications or limited accessibility, MRI is not feasible on every patient or cannot be done as soon as possible after trauma. Consequently, in the event of a negative CR but a significant likelihood of a fracture, CBCT should be taken into Multislice Computed Tomography (MCT) consideration as an alternative imaging modality when evaluating complex anatomic locations involving several overlaid bones, such as the foot and wrist (Figure 3). An MRI may not be necessary later on if fractures are evaluated promptly and accurately. Weight-bearing CBCT offers details on joint alignment in cases of suspected joint instability, but it can only be carried out on specialized CBCT equipment [15,17].

CR may be challenging for callus formation and bone healing follow-up, particularly if there are casts or splints covering the affected area. Compared to CR, CBCT can offer more precise information about bone architecture, which is helpful in assessing the course of recovery, which CR can overestimate or underestimate [19].

## 3. Role of CBCT in Facial Trauma

### 3.1. Mandibular Bone Fractures

Symphyseal–parasymphyseal, body, angle, and ramus fractures are among the anatomic regions that are used to classify mandibular fractures. The two types of fractures that affect the ramus mandible are coronoid process fractures and condylar fractures. Fractures are categorized based on whether they include the mandibular angle, the ascending ramus, or only the dental arc [20]. The condyle, body, and angle are the most common fracture sites in the mandible, while the ramus, parasymphyseal region, alveolar crest, and coronoid process are the least common [21].

Panoramic radiography and standard projection imaging techniques, including transcranial, posteroanterior, and occlusal radiographs, lateral oblique views, and submentovertex skull views, form the basis for evaluating patients with suspected mandibular fractures [22]. Conventional projection radiography, on the other hand, produces a 2D image of a 3D object and has several drawbacks, including the blurring, distortion, and superimposition of anatomical frameworks. Three-dimensional reconstruction without superimpositions is possible with 3D imaging techniques like CBCT, which can produce images in the sagittal, coronal, and axial planes [23]. A radiolucent line, a change in the structure’s typical anatomic outline or shape, a defect in the outer cortical boundary, and an increase in bone density—which could be the result of two bone fragments overlapping—are radiographic indicators of a mandibular fracture [24].

**Figure 4:**
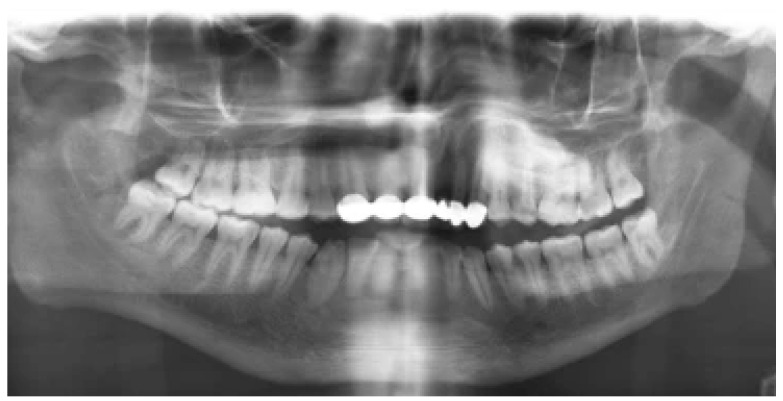
A panoramic radiograph. The symphyseal–parasymphyseal fracture is obscured by the superimposition of cervical vertebrae, and the condyle fracture is obscured by a partial image due to improper patient positioning. Published with permission from [28]. Copyright 2013, Elsevier.

When it comes to identifying fractures in the anterior part of the mandible, such as those in the condylar and coronoid bones, CBCT is more effective than panoramic radiography. The maxillary tuberosity, zygomatic procedure, and the pterygoid procedure of the sphenoid bone can all be used to superimpose the mandibular condyle on panoramic radiographs [25]. The diagnostic performance of Multislice CT (MCT) and CBCT for sheep mandibular condyle fractures was compared in another study. They concluded that when it comes to fracture diagnosis, CBCT and CT are equally accurate [26]. The diagnostic efficacy of CBCT pictures was assessed and compared to conventional images acquired from patients with craniofacial injuries. According to their assertion, CBCT considerably enhances the detection of fracture extensions in the mandibular condylar region and midface when compared to standard radiography [27]. A study investigating the diagnostic capability of CBCT for mandibular fractures has shown that CBCT provides more precise information compared to panoramic radiography in persons with suspected mandibular fractures. Figure 4 illustrates how the condyle and rupture line in the symphyseal–parasymphyseal area can be seen on the pseudo-panoramic view; the poor positioning of the patient when performing the orthopantomography limits the analysis of the exam and, in turn, the comparison with the CBCT at this level may not be fair due to the poor performance of the exam on the panoramic radiograph [28].

The preferred imaging modality for mandibular trauma in cases of suspected mandibular fractures is either CBCT or CT imaging, due to their comparable abilities to assess bony components and provide sufficient 3D hard tissue data. The inability of CBCT to display soft tissue differentiation is a drawback. MRI must be taken into consideration for providing soft-tissue visualization when soft-tissue injury to the articular disc or temporomandibular joint capsule is suspected [29,30].

In addition to 3D reconstructions and images in orthogonal planes, CBCT data can be reorganized, and multiple kinds of visuals in curved or oblique image layers can be synthesized. Sectional image slice thickness and slice interval can be specified. A planning line is also automatically drawn when multiple nodes are selected along the centerline that corresponds to the jaw arch on a suitable axial image. This produces a “simulated” or “pseudo-panoramic” image that is helpful for evaluating the jaw. To see the anatomical framework and prevent the missing of diseases, the pseudo-panoramic image’s thickness is crucial [31,32].

### 3.2. Dentoalveolar Fractures

Any level of the tooth’s root may be affected by a root fracture, which typically occurs in a horizontal, diagonal, or vertical plane. The degree of fragment separation and the angle of the radiation beam with respect to the fracture plane determine whether or not a radiographic image can identify the existence of a root fracture. Conventional radiographs show a single, well-defined radiolucent line if the X-ray beam penetrates the fracture plane. On the other hand, if the X-ray beam is pointed in a reverse direction towards the fracture plane, it can be missed or appear as a less distinct single line that converges at the distal and mesial edges of the root [33]. The absence of pathognomonic signs and the variety of clinical presentations can make diagnosing a root fracture challenging in certain cases. A localized enlargement of the periodontal ligament space next to the fracture site may be the only sign of a fracture [34].

The studies evaluating the effectiveness of CBCT in the identification of root fractures were made possible by the challenges associated with identifying root fractures in practice and with the use of conventional radiography. Long and his colleagues’ meta-analysis sought to ascertain CBCT’s diagnostic accuracy for tooth fractures in vivo. They discovered that the combined sensitivity and specificity were 0.92 and 0.85, respectively [35]. A meta-analysis and systematic review focusing on the precision of root fracture detection in CBCT images of teeth without fillings found that the i-CAT^®^ CBCT unit had a pooled sensitivity of 0.83 and a pooled specificity of 0.91. For the 3D Accuitomo CBCT unit, the pooled sensitivity was 0.96 and the pooled specificity was 0.95 [33]. The impact of various voxel sizes on the precision of root fracture detection has also been studied within this scope. A study by Özer found that, given the reduced X-ray exposure and satisfactory diagnostic efficacy, a 0.2 mm voxel was the optimal protocol [36]. According to Da Silveira and her colleagues, the choice of voxel size should be based on the condition of the roots; teeth without fillings should have a voxel size of 0.3, while teeth with fillings and/or posts should have a voxel size of 0.2 [37]. In the research carried out by Bragatto and his colleagues, the identification of vertical root fractures could be done with voxel sizes of 0.200 mm and 0.250 mm with 100% and 90% accuracy, respectively [38]. The 0.100 mm voxel size (without an optimization filter) has been suggested by Parrone and colleagues for the detection of root fractures in teeth that have undergone endodontic treatment. While individual studies offer valuable insights, meta-analyses and systematic reviews have produced inconsistent findings regarding the body of available evidence. The significance of appropriate voxel size is emphasized by an in-depth investigation and meta-analysis [39]. In particular, when a voxel size smaller than 0.2 mm is used, CBCT has a substantially greater sensitivity than periapical radiography, in accordance to the in vivo studies that were examined in the study [40,41]. However, a study concluded that, in teeth that are not filled with roots, voxel size has no bearing on the diagnostic efficacy of root fracture. According to the authors, more research is still needed to determine the diagnostic reliability of root fractures in teeth that have posts or that are filled with roots. Regrettably, the diagnostic precision of root fractures in CBCT scans is reduced by both metal artifacts and artifact-reduction algorithms [42]. As a result, in addition to the type of imaging examination, the presence and kind of materials used for the tooth’s restoration and root canal therapy also affect the identification of vertical root fractures [43]. A systematic review on the effect of the size of voxel in CBCT-based image acquisition, however, came to the conclusion that there is currently no universal protocol that can be established for CBCT examination of particular dental diagnostic tasks, such as root fracture detection [44]. Furthermore, since the physical voxel size is only one of many parameters that affect spatial resolution—others include scatter, motion blur, the 2D detector, and the 3D reconstruction process—the actual spatial resolution will always be significantly smaller than the voxel size [9].

An associated root fracture may also be present in an alveolar fracture, which is defined as a fracture of the alveolar process. It can involve the lingual or buccal cortical plates regardless of the concurrent involvement of the alveolar socket. It may be challenging to distinguish between a root fracture and an adjoining fracture line of alveolar bone on intraoral radiographs if there is a single cortical wall fracture of the alveolar process [45].

Because CBCT has greater diagnostic precision than typical radiographs, it is advised for the recognition of dentoalveolar fractures [46]. Avsever et al.’s study examined the effectiveness of CBCT and intraoral radiography in identifying horizontal root fractures. They recommended that the most dependable imaging technique for confirmation of horizontal root fractures is CBCT [47]. 

**Figure 5:**
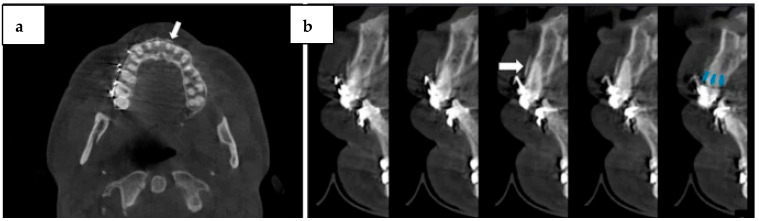
(**a**) Longitudinally displaced cortical plate fracture (arrow) is visible in the axial CBCT image. (**b**) Alveolar fracture (white arrow) and radiolucent artifact from metal brackets mimicking a crown fracture (blue arrows) are seen in cross-sectional CBCT images. Published with permission from [34]. Copyright 2021, Elsevier.

The cross-sectional plane images provide detailed information about the number, orientation, and extent of fractures. The axial section of the labially displaced cortical plate fracture is depicted in Figure 5a. Nevertheless, streaking image artifacts from dense objects like metal posts and brackets, root canal filling material, and the inability to separate fracture fragments make root fracture detection on CBCT still difficult. Because of the beam-hardening effect, these artifacts resemble fracture lines and show up as dark bands or streaks. The artifact on cross-sectional CBCT scans caused by metal brackets is displayed in Figure 5b [34].

### 3.3. Facial Fractures

With studies showing improved implant positioning, fracture reduction, and decreased need for a second operation, especially in situations involving articular fractures or breaks involving intricate anatomical areas, intraoperative 3D imaging initially acquired traction in orthopaedic surgery [48].

The use of intraoperative 3D imaging in maxillofacial trauma has increased over the past ten years. In early feasibility studies, Manson noted that intraoperative medical evaluation for sufficient decrease in orbital reconstructions and zygomatic complex fractures was frequently not verified by the post-operative computed tomographic (CT) scan, even among highly skilled surgeons. By enabling quick assessment of implant or bone positioning, intraoperative CT can reduce the chance of additional surgical revision [49]. 

Following surgical correction of ZMC fractures, Pohlenz and colleagues reported the first clinical uses of intraoperative CBCT with a coupled flat-panel detection system in oral and maxillofacial surgery [50]. Comparing the C-arm CBCT to a medical-grade CT, it takes up less room and is thought to be easier to operate. Including setup, sterile draping, and image review, the additional surgical time was estimated to take 8 to 30 min [51]. The FOV of this particular CBCT C-arm is limited to 12 cm × 12 cm × 12 cm, which means it cannot image the contralateral side. To accomplish this comparison, Assouline and colleagues recommend combining the intraoperative information with pre-operative imaging [52].

Applications include orbital reconstructions and the evaluation of decreases in zygomaticomaxillary fractures (ZMC). Since direct visualization is not possible with surgical approaches, ZMC reduction is typically evaluated by subjectively assessing facial symmetry and sphenozygomatic suture reduction. Intraoperative CBCT enabled for immediate revision in 6 out of 48 cases in a retrospective study of 48 patients with orbit fractures or zygoma, especially in cases of comminution [53]. Moreover, it was determined that there was no longer a demand for orbital exploration in seven patients after Zygomaticomaxillary complex (ZMC) reduction, which avoided needless orbital exploration. A level 1 trauma centre in Portland, Oregon conducted a study on intraoperative CT, and the results showed that the following CT-guided revision rates applied: Zygomaticomaxillary complex 24%, orbital 31%, Le fort I 8%, naso-orbital ethmoid 23%, Le fort II and III 23%, frontal sinus 0% and mandible 13% [54].

## 4. Role of CBCT in Dental Disorders

### 4.1. Endodontics

Since caries identification and depth assessment in occlusal and approximal lesions have been substantially enhanced, CBCT can also be used for diagnosing caries. On the other hand, using it in endodontic-metallic restorations would result in artifacts that lower diagnostic precision. Only unrestored teeth should be CBCT-imaged for caries. With CBCT, sensitivity may rise, but specificity shouldn’t suffer in the process. Using grayscale values found in the lesions, CBCT in endodontics can be used to distinguish solid from fluid-filled lesions (such as periapical granulomas from cysts) and to identify and measure the scale of periapical lesions [55]. Through the identification of every root canal, CBCT contributes significantly to the establishment of efficient endodontic therapy by facilitating access, cleaning, shaping, and obturation of each canal. It aids in determining the frequency of multiple and accessory canals (aberrant pulpal anatomy, such as dens invaginatus) in any other teeth as well as the second mesiobuccal canal (MB2) in maxillary first molars [56]. Additionally, it facilitates the distinction between pathosis and normal anatomy as well as the relationships with key anatomical structures. In addition, CBCT is crucial for the diagnosis and treatment of alveolar displacements, fractures, or luxation and root fractures [57]. 

### 4.2. Orthodontics

When evaluating the orientation of unerupted teeth, especially impacted ones, an orthodontist can use CBCT. This is especially true for maxillary impacted canines, where precise tooth positioning is necessary for successful therapy planning. If the photographs were taken in two distinct planes, the angulation would be difficult to see on a conventional radiograph, but it is well appreciated in these three-dimensional images. Additionally, it aids in the detection of any neighboring tooth resorption (i.e., ectopic maxillary canines and suspected resorption of the incisor roots) [58].

Assessment of cleft palate, resorption associated with rapid maxillary expansion, impacted teeth, surface imaging integration, 3D cephalometry, age assessment, airway assessment, and research into orthodontic-associated paranesthesia are additional uses of CBCT in orthodontics [59]. To reduce complications, CBCT is used to assess bone dimensions and the exact placement location of mini-screw implants for anchorage [60].

### 4.3. Periodontics

CBCT in periodontics measures bone quantity, craters, furcation, and loss of crestal bone, fenestrations, and dehiscence. Despite being helpful in this area, CBCT is not recommended as a standard technique for visualization of periodontal bone support. However, in particular instances of infrabony imperfections and furcation tumors, where medical and traditional radiographic examinations fail to yield the necessary information for management, restricted-volume, excellent quality CBCT may be indicated. As a result, it may be useful in dealing with intricate periodontal imperfections for which surgery is the recommended course of treatment. In addition, within a month, CBCT can be used in place of subtraction radiography for post-operative imperfection fill or bone strength assessment, which are not detectable with standard radiographs [61].

By measuring gingival tissue, evaluating periodontal ligament space, and determining the size of the dentogingival unit, CBCT can assist in determining the size of bone defects and early indications of periodontitis [62]. This technique, known as ST-CBCT, aids in the accurate visualization and measurement of distances that correspond to the periodontium’s and the dentogingival attachment apparatus’s hard and soft tissues. Gingival margin and the crest of the facial bone, gingival margin and the cementoenamel junction (CEJ), and CEJ and the crest of the facial bone can all be identified with ST-CBCT [63,64].

### 4.4. Current Trend of CBCT in Periodontology Therapies

Because CBCT is so common, new studies are showing that it can be used to treat periodontal disease. Despite the paucity of data, what is known suggests that CBCT may play a bigger part in periodontal therapy. This is the area where sophisticated radiographic examination and post-surgical evaluation currently have the strongest confirmation supporting the application of CBCT in periodontal therapy. Completing a clinical assessment (such as suppuration/bleeding on probing, probing depths, attachment level, furcation involvement, mobility etc.) and a radiographic assessment (periapical and bite-wing photos) has been and still is the benchmark for periodontal examination. When it comes to diagnosing periodontal disorders involving the existence of intrabony imperfections and furcation, the traditional examination has long been the most reliable. While clinical assessment and a standard 2D intraoral radiography exam (IOR) are effective methods for identifying the existence of intrabony problems and furcation involvement, there is growing evidence that CBCT imaging can be a useful adjunct to the diagnostic process [65].

According to Braun and his colleagues, CBCT is a better method than IOR for identifying intrabony problems and furcation involvement. In total, 82.7% of intrabony defects were correctly identified using IOR, and 99.7% were identified using CBCT. Additionally, compared to IOR (75.6%), CBCT was more effective at detecting furcation involvement (94.8%). They concluded that the third dimension greatly improved the CBCT diagnostic accuracy [66]. When comparing IOR versus CBCT, Brags and his colleagues found similar results for the identification of fenestration (25.7% vs. 89.1%) and dehiscence (46.8% vs. 78.2%) [67]. Similar conclusions have been reached by a number of other studies: inter-examiner agreement is higher with CBCT than with IOR, CBCT is more precise in detecting intrabony malformations and furcation activity, and IOR typically underestimates the seriousness of each [68,69].

Walter and his colleagues conducted a study to assess the reliability of traditional testing, which involves clinical examination with intraoral radiographs (IORs), compared to the use of additional cone-beam computed tomography (CBCT) evaluation. The aim was to determine the extent of furcation involvement and the appropriateness of treatment planning for maxillary molars with furcation involvement. Maxillary molars were evaluated after the initial non-surgical treatment was finished. This involved gathering standard periodontal data and classifying furcation involvement using a curved Nabers probe to detect furcation involvement in accordance with Hamp’s classification. IORs supported the clinical evaluation, and a plan of care was established. Subsequently, CBCT assessments were finished, examined to ascertain the extent of furcation engagement, and contrasted with the results of the traditional evaluation [70].

Researchers found that conventional assessment accurately determined the degree of furcation participation just 27% of the time, with the highest accuracy observed for degree III furcation. The degree of participation was overestimated 29% of the time and overlooked 44% of the time in conventional assessments. Most of the time, degree II furcations were taken for granted and degree I furcations were overestimated. Additionally, they discovered that 41% of the time, the suggested treatment plan based on the CBCT data and that based on the conventional assessment agreed. In other cases, the treatment regimens selected by the conventional assessment were shown to be 18% less invasive and 41% more invasive than when relying on CBCT data. The researchers determined that using CBCT data improved the assessment of furcation involvement and facilitated the development of a more appropriate treatment plan [70].

Further research has determined that furcation engagement, intrabony periodontal imperfections, and horizontal bone loss can be accurately measured and identified using CBCT evaluation. According to Juerchott and colleagues, while comparing CBCT evaluation with intra-surgical findings, it was shown that CBCT accurately measured maxillary molar furcation involvement 95% of the time. It underestimated 1.7% of the time and only overestimated 0.39% of the time [71]. According to Padmanabhan and his colleagues, there was no statistically significant difference in furcation height, width, and depth between direct intra-surgical measurements and CBCT [72]. As per Banodkar and colleagues, CBCT is exceptionally accurate in assessing the vertical levels of abnormalities and highly exact in detecting the specific type of periodontal defect [73]. 

Zhao and his colleagues reported that another advantageous use of CBCT was its capacity to evaluate the root concavities of first premolars and the corresponding pattern of bone loss. Based on the origin of the concavity, they classified roots into five categories. The onset of Type II concavity occurred in the enamel layer, with Type II being aligned with the cementoenamel junction (CEJ). Type IV was situated below the CEJ, namely in the upper two-thirds of the root, while Type V was found in the lower third of the root. Type I lacked concavity. The pattern of bone loss was defined as either a ramp, flat, or crater. Maxillary first premolars consistently exhibited mesial concavities, with Type II being the most prevalent, occurring in 35.7% of cases. Only 39.3% of the sites had a distal concavity, with Type IV being the most prevalent, accounting for 14.2%. Mandibular first premolar concavities were much less common; only 42.5% of the mesial (Type II–V evenly distributed) and 31.3% of the distal (Type IV most common, 15%) of the premolars were found to have concavities. It was interesting to see how the trends of bone loss were distributed. When separated by the existence or lack of a concavity, they exhibited consistency between the distal and mesial sites. When a concavity was present in the mesial sites, a crater, ramp, and plane were observed 37.8%, 3.17%, and 30.6%, respectively, whereas when a concavity was absent, the corresponding values were 14.1%, 58.7%, and 27.2%. The distribution of the distal sites was very similar: at sites with a concavity, they were 40.4%, 31.9%, and 27.7%, respectively, while at sites without one, they were 14.0%, 57.9%, and 28.1%, respectively [74].

This level of detail is frequently not described or certain according to traditional evaluation. An improved prognostic assessment and therapy plan can be achieved with more precise information available. The systematic review conducted by Nikolic-Jakoba et al. determined that there were insufficient data to support the use of CBCT for identifying intrabony and furcation abnormalities. Although this conclusion might seem depressing, it was primarily brought about by the paucity of scientific literature available at the moment. They added that, in situations where traditional assessment may fall short, it may still be clinically advantageous to use CBCT [75].

Despite the paucity of data, certain clinical scenarios are starting to emerge where CBCT could prove to be a useful supplement to traditional evaluation. In addition to the benefits, CBCT can enhance treatment and surgery planning in various ways. When strategizing surgical treatments, CBCT can assist a clinician in locating and delineating significant anatomical features such as the larger palatine, lingual, inferior alveolar, or mental nerve [76,77]. By measuring the alveolar process’s hard and soft tissue thickness, CBCT can be used to assess the biotype. In addition, it can be used to identify fenestrations over root surfaces and to measure the thickness of the facial plate. These data can be beneficial for surgical preparation, specifically for complex augmentation of soft and/or hard tissues, as well as for diagnosing altered passive eruption based on the relationship between the crestal bone level and the teeth [78,79]. However, post-treatment evaluation is one of the most beneficial uses of CBCT assessment.

In addition to its accuracy in identifying and measuring intrabony flaws and furcation involvement, as discussed earlier, CBCT imaging can also be used to precisely assess bone levels using circumferential estimates and a typical six-site approach [80]. The American Academy of Periodontology (AAP) Regeneration Workshop in 2015 specifically advised the use of CBCT evaluation following regenerative surgical treatment for furcation involvement [81].

AAP published a series of papers titled "Best Evidence Consensus (BEC)" that discuss the use and application of CBCT in periodontics. A team of specialists, highly knowledgeable and experienced in the application of CBCT, was convened to conduct a comprehensive literature study and generate a consensus statement regarding its various uses [82]. It was determined that there are insufficient data to support the use of CBCT as the standard of care for the diagnosis, preparation, and treatment of periodontal disease. Although CBCT imaging can be beneficial in certain cases, such as advanced intrabony and furcation defects and suspected endo-perio lesions, traditional evaluation methods were still considered to be the most reliable. The clinician is responsible for implementing the “As Low As Reasonably Achievable” (ALARA) guideline and assessing the utility of CBCT imaging Although most CBCT contacts are low-exposure, the ALARA principle would not be followed if the data from an extra CBCT were not helpful for diagnosis, prognosis, or treatment management [83].

### 4.5. Dental Implant Placement

A significant portion of humanity suffers from either complete or partial dentition. It impairs the sufferer’s quality of life and makes it challenging to keep one’s health at its best. Because of the shortcomings of 2D techniques, CBCT has gained much traction and, because of its many benefits, is now an essential component of dental implantology. The creation of multiplanar sections and the 3D reconstruction of structures are its primary strengths. To guarantee the procedure’s success, it also permits an evaluation of bone quality and density [84]. 

Dental implant planning and placement relied on clinical assessments, 2D radiological techniques, and dental study analysis prior to the development and widespread application of CBCT. A few of the shortcomings of the aforementioned techniques are magnification error, distortion, and structural superimposition. A precise and accurate determination of the separation among neuro-vascular and anatomical structures and dental implants cannot be achieved using 2D methods [85]. The use of CBCT in dentistry is growing in popularity these days because of its high quality, analytical potential, and the accurate data it offers. Dental implants are now the method of choice for treating physical trauma, ailments, unusual tooth growth and development, and aesthetic concerns, due to their longevity and high quality. A few benefits of CBCT that have cemented its spot in dental implantology are its high resolution, low radiation doses, accuracy, and cost savings for the patient [86]. Surgery using CBCT takes fewer minutes and results in less pain and swelling after surgery. It comprises sagittal, lateral, axial, and coronal cephalometric projections, and 3D and 2D panoramic projections. Its value extends beyond implant planning and placement, as it can be utilized to identify and examine pathological processes in dentistry, including endodontic infections, benign or malignant tumors, cysts, and dental caries [85]. 

## 5. Comparison of CBCT with Conventional Technologies

The high spatial resolution of CBCT, which produces exquisite detail of the bone microarchitecture, is one of its main advantages. CBCT arthrography, also known as CBCT-A, allows for the acquisition of high-resolution pictures of the surface of the articular cartilage after injecting contrast into the joint [87]. The spatial resolution achievable with our apparatus varies from 300 μm for a typical scan to 75 μm for images with a high resolution. Current research validates that the dose of CBCT is significantly lower than that of MDCT, both in a cohort of pediatric patients and on phantoms [88]. About 40% less is used in CBCT than in standard MDCT, and 30% less is used in low-dose sinus CT scans for paranasal sinus imaging. Research conducted on ankle phantoms revealed an effective dose of 21.4 μSv for MDCT, while 1.9 μSv to 14.3 μSv was reported for CBCT. Using a large, high-quality flat-panel detector, a narrower field of sight, pulsed X-ray beams instead of continuous radiation, and a single rotation of the gantry to capture the full scan volume results in decreased radiation exposure [89].

Our NewTom 5G equipment incorporates SafeBeam technologyTM, which effectively minimizes radiation exposure despite utilizing a fixed tube voltage of 110 kVp. Once the patient’s size has been estimated using attenuation data gathered from anteroposterior and lateral scout views, the tube current (mA) is then optimized. The X-ray tube rotates around the patient in the anteroposterior and lateral positions, adjusting its position based on the calculated reduction from the previous projection. Angular tube current modulation then equalizes the photon flux to the detector in real time, allowing for further customization of the dose based on the patient’s anatomy [90].

Instead of continuously emitting radiation over a full 360-degree rotation, pulsed emission technology limits exposure to irregular bursts of radiation for each degree. As a result, the efficient time of exposure is significantly reduced (for example, if a 360° rotation takes 18–36 s, the operational exposure time is only 2.4–7.3 s). This has no effect on the overall quality of the image because qualitative image reconstruction can be done using datasets from 360 projections—one for each degree of 360° rotation [91].

When applying a conversion factor of 0.01 mSv/Gy × cm^2^ to peripheral joints, the effective dose (ED) spectrum in CBCT for small joints varies from 1–15.3 μSv. This is far lower than the values documented for MDCT. Conventional radiography (CR), which uses radiation, continues to have the lowest ED of all imaging techniques (between 0.07 and 5 μSv) [16].

However, implanted metal elements may change the visual appeal of CT and CBCT images by decreasing contrast, obfuscating structures, and making it harder to identify areas of interest. By employing specialized software and algorithms, this picture loss may be lessened [92]. Since the equipment is significantly less expensive overall than MDCT, it can be used as supplemental CT equipment in larger institutions or in private practices or small medical centres. A summary of the factors that could affect how economically rentable it is to install a CBCT is given in Table 1. 

Nevertheless, it is impossible to make broad recommendations because referral patterns for specific tests may vary amongst medical facilities and reimbursement may vary greatly based on national health policies. One drawback of CBCT is its narrow FOV, which with our apparatus can be as little as 6.6 cm and as much as 18.16 cm. As a result, large joints cannot be imaged using CBCT. Another disadvantage is that Hounsfield Unit (HU) measurements and lack of contrast resolution make it difficult to evaluate soft-tissue pathology. Moreover, CBCT requires short acquisition times, which increases its vulnerability to motion artifacts [93]. The summary of primary benefits and drawbacks of musculoskeletal CBCT are enumerated in Table 2.

## 6. Future Recommendations

Recent flat-panel detector C-arm units can provide guidance for interventional radiation therapy procedures by combining fluoroscopy and CBCT imaging [94]. Nucleoplasty, vertebroplasty, and bone biopsies are among the prevalent percutaneous interventional musculoskeletal procedures. Imaging guidance is being used for palliative or ablation operations, including CBCT [95]. The CBCT application can aid in determining the optimal course of action, accurate monitoring, and identification of errors in the operating room when achieving precise positioning of the biopsy needle is difficult. Utilizing CBCT guidance can decrease fluoroscopy duration, leading to less cumulative radiation exposure for both patients and staff [96]. Additionally, reports of fusion with MRI for accurate lesion targeting have been made [97]. Under MDCT and CBCT guidance, the procedures take about the same amount of time overall, and for CBCT, the patient and the performing physician receive reduced doses. It is crucial to use a lower radiation dose, particularly in younger patients. There are other systems available that can be used to assess joint stability during weight-bearing extremity exams [98]. Every business case for buying CBCT equipment should be customized to the unique requirements in each department due to the unique design (gantry size, light guide for needle position) for each application.

## 7. Conclusions

Unquestionably, CBCT has revolutionized the fields of musculoskeletal, dental, and maxillofacial imaging by offering previously unobtainable insights into dental disorders, bone abnormalities, and facial trauma. Its capacity to generate three-dimensional, high-resolution images has significantly improved treatment planning and diagnostic accuracy. However, care must be taken to manage CBCT’s drawbacks, which include radiation exposure, expense, and the requirement for specialized training. Future developments and standardized procedures will be essential to optimizing CBCT’s advantages and reducing its disadvantages. Through the resolution of these issues and the pursuit of ongoing innovation, CBCT can strengthen its position as a vital instrument in contemporary dentistry, maxillofacial, and musculoskeletal practice. A prudent and knowledgeable application of CBCT, informed by ongoing research and advancements in technology, will guarantee seamless integration into clinical workflows, ultimately enhancing patient outcomes and care.

## Figures and Tables

**Table 1:** Comparison of factors affecting CBCT versus MDCT’s economic rentability.

Parameter	CBCT	MDCT
Initial Equipment Cost	Usually Lower	Generally higher because of complex technology
Maintenance Cost	Lower	Higher
Radiation Dose	Lower	Higher
Image Quality for Bone Structures	High (sufficient for dental/maxillofacial)	Very High (detailed cross-sectional images)
Image Quality for Soft Tissue	Limited	High (excellent soft tissue contrast)
Scan Time	Short (few seconds)	Variable, usually longer
Operational Training Requirement	Moderate	High due to complexity
Patient Throughput	High (quick scans, efficient workflow)	Moderate (longer scan and processing times)
Space Requirements	Smaller footprint (16.5 m^2^–33 m^2^)	Larger footprint (36 m^2^–52 m^2^)
Versatility	Limited (primarily dental/maxillofacial)	High (broad applications across body)
Reimbursement Rates	Generally lower	Higher due to broader applications
Overall Cost per Scan	Lower	Higher
Patient Comfort	Higher (shorter and quieter scans)	Moderate
Availability and Accessibility	High in dental practices	High in hospitals and imaging centres
Regulatory Requirements	Moderate	High due to radiation dose and application

**Table 2:** Benefits and drawbacks of musculoskeletal CBCT in comparison to conventional techniques.

Parameter	CT	CBCT	MDCT	CR	MRI
Primary Use	Broad diagnostic imaging	Detailed bone imaging, dental/maxillofacial	Comprehensive body imaging	Basic bone imaging	Soft tissue and bone imaging
Versatility	High (multiple applications)	Limited (primarily musculoskeletal, dental)	Very high (multiple applications)	Limited (primarily bone)	Very high (multiple applications)
Image Resolution (Bone)	High	High (excellent for bone structures)	Very high	Moderate	Moderate
Radiation Dose	Higher than CR, lower than MDCT	Lower than CT/MDCT	Higher than CR and CBCT	Very low	None
Space Requirements	High	Moderate	High	Very low	High
Artifacts	Moderate	Moderate (metallic artifacts common)	Moderate (metallic artifacts common)	Minimal	Susceptible to metal and motion artifacts
Operational Training	High	Moderate	High	Low	Very high
Patient Comfort	Moderate	High (quick and quiet scans)	Moderate	High	Low (longer, noisy scans)
Suitability for Implants	Good	High	High	Poor	Moderate (depending on the implant)
Scan Time	Moderate	Short (few seconds)	Moderate	Very short (seconds)	Long
Image Resolution (Soft Tissue)	Moderate	Limited	High	Poor	Excellent
Cost	High	Moderate	High	Low	Very high
Availability	High	Increasing in dental practices and clinics	High	Very high	High
Detailed Bone Structure	Excellent	Excellent (3D imaging)	Excellent	Limited (2D imaging)	Good
Soft Tissue Contrast	Moderate	Limited	High	Poor	Excellent
Patient Throughput	Moderate	High (quick scans, efficient workflow)	Moderate	Very high	Low (longer scan times)

## List of Abbreviations

CT	Computed Tomography
CBCT	Cone-Beam Computed Tomography
3D	Three-Dimensional
2D	Two-Dimensional
MDCT	Multidetector Computed Tomography
MRI	Magnetic Resonance Imaging
ALARA	As Low as Reasonably Achievable
MTF	Modulation-Transfer Function
CR	Computed Radiography
mm	Micrometer
ZMC	Zygomaticomaxillary Complex
IOR	Intraoral Radiography
CEJ	Cementoenamel Junction
AAP	American Academy of Periodontology
BEC	Best Evidence Consensus
ED	Effective Dose
CR	Conventional Radiography
FOV	Field of View
